# Identifying causal relationships of cancer treatment and long-term health effects among 5-year survivors of childhood cancer in Southern Sweden

**DOI:** 10.1038/s43856-022-00081-z

**Published:** 2022-03-02

**Authors:** Anders Holst, Jan Ekman, Magnus Petersson-Ahrholt, Thomas Relander, Thomas Wiebe, Helena M. Linge

**Affiliations:** 1 Department of Computer Science, Division of Digital Systems, Research Institutes of Sweden, Stockholm, Sweden; 2 Climber, Malmo, Sweden; 3 Department of Clinical Sciences Lund, Oncology, Skane University Hospital, Lund University, Lund, Sweden; 4 Department of Clinical Sciences Lund, Pediatrics, Faculty of Medicine, Lund University, Lund, Sweden

**Keywords:** Paediatric cancer, Databases

## Abstract

**Background:**

Survivors of childhood cancer can develop adverse health events later in life. Infrequent occurrences and scarcity of structured information result in analytical and statistical challenges. Alternative statistical approaches are required to investigate the basis of late effects in smaller data sets.

**Methods:**

Here we describe sex-specific health care use, mortality and causal associations between primary diagnosis, treatment and outcomes in a small cohort (*n* = 2315) of 5-year survivors of childhood cancer (*n* = 2129) in southern Sweden and a control group (*n* = 11,882; age-, sex- and region-matched from the general population). We developed a constraint-based method for causal inference based on Bayesian estimation of distributions, and used it to investigate health care use and causal associations between diagnoses, treatments and outcomes. Mortality was analyzed by the Kaplan–Meier method.

**Results:**

Our results confirm a significantly higher health care usage and premature mortality among childhood cancer survivors as compared to controls. The developed method for causal inference identifies 98 significant associations (*p* < 0.0001) where most are well known (*n* = 73; 74.5%). Hitherto undescribed associations are identified (*n* = 5; 5.1%). These were between use of alkylating agents and eye conditions, topoisomerase inhibitors and viral infections; pituitary surgery and intestinal infections; and cervical cancer and endometritis. We discuss study-related biases (*n* = 20; 20.4%) and limitations.

**Conclusions:**

The findings contribute to a broader understanding of the consequences of cancer treatment. The study shows relevance for small data sets and causal inference, and presents the method as a complement to traditional statistical approaches.

## Plain language summary

Survivors of childhood cancer can develop late effects in adulthood. Knowledge about possible late effects can improve childhood cancer treatments and assist in follow-up. We developed a method to identify causative links between treatments and health outcomes. We applied it to a Swedish patient cohort and identified 98 causative links between treatments and outcomes, many of which are already known. Some, however, have not been previously described, including links between certain treatments and eye conditions or viral infections. We also confirm that childhood cancer survivors use more health care and have higher mortality compared to the general population. This study helps to create a better understanding of the late effects of cancer treatment in children and may help to guide strategies to monitor and treat children to avoid these effects.

## Abstract (web summary)

Holst et al. study the relationship between cancer treatments and long-term health effects using registry data on childhood cancer survivors in Sweden. The authors utilize a causal inference approach to establish relationships between certain therapies and viral infections, eye conditions, and reproductive conditions, amongst others.

## Introduction

Survival after childhood cancer has increased and resulted in a growing population of survivors at risk for developing late complications^1,2^. The most apparent late effects have been clinically evident since the beginning of the combinational therapy era in the 1970s and have in recent years been transformed into evidence-based guidelines to aid medical follow-up^3–6^. Extensive studies have resulted in risk associations between adverse outcomes, e.g., cardiovascular events^7^, impaired cognition^8^, and specific chemotherapeutic drugs, targets and doses of radiation therapy as well as stem cell transplantation^9^. In the current study, we developed a method for causal inference and used it to investigate causal relations between details of the primary disease, its treatment history and health-related events that occur 5 years after the first childhood cancer diagnosis (CCD), using a population-based childhood cancer cohort with years of diagnoses ranging from 1970 to 2016^10^. The purpose of the study was to (1) briefly describe the newly established cohort, (2) describe and discuss the causal inference method as suitable for smaller data sets and (3) report the links found between primary disease, treatment and outcomes. We hypothesized that the analysis would identify novel associations, which may enrich clinical follow-up care of childhood cancer survivors (CCS), and contribute to an improved understanding of the basis of adverse effects after cancer treatment. The study confirmed a higher health care usage and mortality among CCS than in the control group from the general population, and identified 98 links between primary disease, treatment and outcomes. Most of the links were well known but some have not been described previously, to the best of our knowledge. These were between use of alkylating agents and eye conditions, topoisomerase inhibitors and viral infections; pituitary surgery and intestinal infections; and cervical cancer and endometritis. The developed method for causal inference for limited size data sets is a substantial result of this study.

## Methods

### Ethical considerations

Ethical approval of the study was received by the Regional Ethical Review Board in Lund (2018-022) and the treatment data were extracted from the regional pediatric oncology registry BORISS with permission from the Council of Skane. The law governing quality registries does not impose a requirement of consent from the individual. At the time of diagnosis, the parents of the patients were informed and presented with the possibility of opting out of several quality registries. All data in the project were pseudonymized to allow identification by health care services in case life-threatening late effects were identified.

### Data assembly and coding

The demographical data and detailed treatment data of all 5-year survivors in the regional quality registry^10^ were included in the study (*n* = 2315). They were diagnosed with childhood cancer between the years 1970 and 2012. The patients diagnosed with childhood cancer in 2013–2015 were not yet 5-year survivors (*n* = 186) at the time of data extraction. The detailed treatment data from these latter patients contributed to the analyses but their outcomes did not. Matching control subjects from the general population were selected based on sex, year of birth and place of residency, and served as a control group (*n* = 11,882). Although outcome data are routinely recorded in national registries for all Swedish citizens including CCS, detailed and structured childhood cancer treatment data were available only for the regional subset of CCS. For this study, the outcomes data included in-patient care, outpatient care, and causes of death, represented as International Statistical Classification of Diseases and Related Health Problems 10th Revision (ICD-10) codes. The median time of follow-up was 15.1 years and the mean 16.4 years. The outpatient care registrations started in 1997, resulting in a median time of follow-up of 13.3 years and the mean 11.8 years for outpatient data. Treatment data included chemotherapeutic agents (grouped into 10 categories), anatomical site of radiation therapy (10 categories), type of surgery (86 categories), and stem cell transplantation (allogeneic or autologous). Predisposing medical conditions or syndromes (multiple endocrine neoplasia type 2, neurofibromatosis, Down syndrome, Beckwith–Wiedemann syndrome, and tuberous sclerosis) in the CCS cohort were included as a subgroup (*n* = 101).

### Causal inference approach

The analysis approach was to use a constraint-based method for causal inference, the PC algorithm^11,12^ (named after the inventors Peter Spirtes and Clark Glymour), to build a causal graph between all individual properties, childhood cancer diagnoses, cancer treatments, and potential late effects. In order to use the method with a limited amount of data, we developed a specially tailored conditional independence test, based on Bayesian estimation of distributions and a measure of correlation, which is invariant under conditioning. This makes it possible to base the estimation of the correlation on the whole data set also when conditioning on one or more variables, and thus makes it possible to maintain a high level of significance despite a limited amount of data.

Each potential late effect, as given by the register data records, is represented by an ICD-10 code, henceforth termed “outcome diagnosis code” or just “outcome”, and has a certain base frequency of occurrence in the population. We wish to test whether the frequency of a certain outcome is significantly higher for CCS having received specific treatment. We make two model assumptions: first, we assume an exponential distribution of the occurrence of a specific outcome in the population, i.e., the time *t* until the first occurrence of an outcome with frequency λ is distributed as follows:

1 $$P\left(t\right)=\lambda {e}^{-\lambda t}$$

Second, we assume that each treatment or other exposure that may have an impact on an outcome, will independently of the other exposures cause a possible increase Δλ to the base frequency of that outcome. Both assumptions are only approximately true—the first because many outcomes increase in frequency with increasing age^13^, and the second because there may be exposures that instead increase the sensitivity to other exposures, making the different exposures statistically dependent. Justification of the assumptions and discussion of the possible implications of violations to them is included in the Discussion section.

### Derivation of the unconditional independence test

Given the exponential distribution assumption, we can characterize the observations *D* in a population regarding a specific outcome in terms of the number of individuals *n* which had this outcome, and the total observation time *t* of all individuals, up till the first occurrence of the outcome in each individual. Then we can express the Bayesian estimation of the distribution of the frequency λ of the outcome as follows:

2 $$P\left(\lambda {{{{{\rm{|}}}}}}D\right)\propto {\lambda }^{n}{e}^{-\lambda t}$$

where we have assumed a uniform prior over λ.

To calculate whether the frequency in one subpopulation is significantly higher than in another subpopulation, we consider the distribution of the difference between the two frequencies, Δλ. Once we have this distribution we can make a hypothesis test with the null hypothesis that Δλ = 0. First, we express the joint distribution of the two frequencies of the outcome in the two subpopulations, set the first frequency to λ and the second to λ + Δλ, and integrate over λ:

3 $$P(\Delta \lambda |{D}_{1},{D}_{2}) 	= \int _{\lambda }P(\lambda |{D}_{1})P(\lambda +\Delta \lambda |{D}_{2})\\ 	 \, \propto \int _{\lambda }{\lambda }^{{n}_{1}}{e}^{-\lambda {t}_{1}}{(\lambda +\Delta \lambda )}^{{n}_{2}}{e}^{-(\lambda +\Delta \lambda ){t}_{2}}\\ 	 \, \propto \,\left\{\begin{array}{c}{e}^{-\Delta \lambda {t}_{2}}{\,\!}_{1}F_{1}(-{n}_{2},-{n}_{1}-{n}_{2}-1,-\Delta \lambda ({t}_{1}+{t}_{2}))\quad\Delta \lambda \,\ge\, 0\\ {e}^{\Delta \lambda {t}_{1}}{\,\!}_{1}F_{1}(-{n}_{1},-{n}_{1}-{n}_{2}-1,\Delta \lambda ({t}_{1}+{t}_{2}))\hfill\quad\Delta \lambda \, < \, 0\end{array}\right.$$

Here the result of the integral is expressed in the hypergeometric function _1_*F*_1_. With this distribution, we can now calculate both the expected value of Δλ as an estimate of how large the effect is, and the significance of the difference by considering the area of the tail reaching “beyond” zero (that is, the probability that we mistake a positive difference for a negative, or vice versa).

### Derivation of the conditional independence test

To determine whether a detected effect is direct or indirect, we systematically condition on alternative causes. We keep only those effects that remain significant in this process, thus removing all effects that can be explained by other causes. For this, we need a conditional independence test, i.e., it is necessary to check the significance of non-zero Δλ under conditioning on a set of other potential causes. Here the assumption that each cause has an (approximately) additive effect on the frequency comes in. The frequencies themselves may vary between the different subpopulations. However, if we assume that each treatment adds to the frequency of an outcome, then this frequency difference Δλ will be invariant over the subpopulations. Instead of estimating separate distributions for each subpopulation and assess the significance in each of them, we can thus use Bayesian estimation of this common Δλ from all subpopulations, dramatically reducing the loss of significance that would otherwise occur due to conditioning.

More formally, assume that all conditioning factors are collected into a joint variable *G* that can assume *K* different values. That is, there are *K* different subpopulations, each with a different set of values of the factors in *G.* Every subpopulation, corresponding to *k* ∈ *K*, is in turn divided in two parts, *D*_1_^(*k*)^ and *D*_2_^(*k*)^, respectively with and without the cause under observation. Then the conditional distribution of the difference of frequencies between those with and without the potential cause can be expressed as follows:

4 $$P(\Delta \lambda |{D}_{1},{D}_{2},G) 	\propto \,P(\Delta \lambda )P({D}_{1,}{D}_{2}|G,\Delta \lambda )\\ 	 \, = \,P(\Delta \lambda )\prod P({D}_{1}^{(k)},{D}_{2}^{(k)}|\Delta \lambda )\\ 	 \, \propto \,P(\Delta \lambda )\prod P(\Delta \lambda |{D}_{1}^{(k)},{D}_{2}^{(k)})/P(\Delta \lambda )\\ 	 \,= \,\left\{\begin{array}{c}\prod {e}^{-\Delta \lambda {t}_{2}^{(k)}}{\,\!}_{1}F_{1}(-{n}_{2}^{(k)},-{n}_{1}^{(k)}-{n}_{2}^{(k)}-1,-\Delta \lambda ({t}_{1}^{(k)}+{t}_{2}^{(k)}))\quad\Delta \lambda \ge 0\\ \prod {e}^{\Delta \lambda {t}_{1}^{(k)}}{\,\!}_{1}F_{1}(-{n}_{1}^{(k)},-{n}_{1}^{(k)}-{n}_{2}^{(k)}-1,\Delta \lambda ({t}_{1}^{(k)}+{t}_{2}^{(k)}))\hfill\quad\Delta \lambda < 0\end{array}\right.$$

We have once again assumed a uniform prior for Δλ. The expected value correspond to the size of the conditional effect, i.e., the direct effect associated with the cause after having removed the effects of the causes in *G*. The area of the tail of the distribution beyond zero is again used as the significance of the difference.

### Resulting algorithm description

We now have an invariant Bayesian conditional independence test, suitable for the current domain with its limited amount of data, which we can use to identify the causal structure of the domain. This is based on the first two parts of the PC algorithm with a minor modification of the second step to make the algorithm independent on the order of evaluation. The first step is to identify all significant correlations (direct or indirect) from the potential causes (i.e., predisposing factors, first CCD, and childhood cancer treatment) to each outcome using Eq. 3. In the second step, each identified relation is reconsidered when conditioning on combinations of the other identified potential causes, to remove those relations that can be explained as indirect relations via other factors. In more detail, this step identifies the smallest set of potential causes that cannot make each other non-significant by conditioning, but which together renders all other relations non-significant. The resulting causes are assumed to be the direct causal links to the outcome.

The PC algorithm relies on the faithfulness condition, which states that if there is a direct causal link between two variables, there should be a statistical correlation between them. This makes it possible to only condition on potential causes that are themselves correlated with the effect, rather than testing all possible subsets of potential causes. While it is theoretically possible that a set of causal links exactly cancel each other out, such that a cause and its effect appear completely uncorrelated, it is highly unlikely. In this study, where we assume that each cause monotonically increases the risk of a particular effect, such a cancellation of effects is even less likely.

It may occur that no single unambiguous set can be found, but two or more sets of potential causes mutually make each other non-significant. This may happen if, e.g., two treatments are usually given together and thus highly correlated. In this case conditioning on one treatment will make the other one independent of any late effects and vice versa. If this occurs, it is not possible to determine which of the treatments is the actual cause. In this study, if no single unambiguous set of potential causes could be found then all sets of potential causes were noted as alternative explanations, and left for manual domain-specific analysis to determine whether one was more biologically relevant than the other was.

### Analysis

The cohort was analyzed using the described method. A significance level of 0.01 was used in the conditional independence test to find potential causes and to sieve out non-direct correlations. In total 108 treatment components, 51 primary cancer diagnoses (occurring between ages 0 and 18 years), and five predisposing conditions were investigated as potential causes for 1332 diagnostic outcome codes. Outcomes occurring at least 5 years after the first primary malignancy were counted as diagnostic outcomes, but relapses were not. This resulted in 814 significant correlations after the first step, and 274 remaining proposed causal relations after the second step. Of the 274 relations, 146 were at a significance level in the interval 0.001–0.01, 32 at a significance level in the interval 0.0001–0.001, and 98 at a significance level in the interval 0.0–0.0001. Only the last interval was considered sufficiently significant (see Supplementary Methods “Choice of significance level” for further motivation of this choice), and the resultant 98 relations were taken for validation.

Mortality rates were analyzed according to the Kaplan–Meier method^14^. The frequency of the presumed novel associations was calculated by dividing number of individuals by the accrued number of person-years for the control population, CCS without the specific outcome code, and CCS with the specific outcome code, respectively.

### Validation of results

The results were graded together with medical expertise and extensive literature searches into classes: Category A: previously known or reported effect, Category B: previously unknown effect, and Category C: other; further divided into: C1: probable bias in registering, C2: probable bias due to increased surveillance or awareness after the first cancer, C3: data quality and congregation issues, and C4: suspicion of time era as a confounding factor.

As the pursuit of confirmations of actual outcomes in medical charts was not possible, the 98 associations were tested for reliability by determination of time of diagnosis code registration. Associations that (1) preceded the CCD or (2) besides occurring 5 years post CCD also occurred within the period 0.5–5 years post CCD were identified. We chose 0.5 years post CCD to exclude acute effects of the induction phase of childhood cancer treatment. A subset of associations contained ICD-10 chapter C or chapter D outcome codes as consequences of a pediatric malignancy and was scrutinized for the source of the outcome code. The associations where the outcome codes originated solely in the outpatient setting are indicated, as the reliability of these outcome codes is more uncertain. A significance level of 0.001 was used to verify whether an association remained intact during the additional tests controlling for the factors time and source of registration code.

### Reporting summary

Further information on research design is available in the Nature Research Reporting Summary linked to this article.

## Results

**Fig. 1:**
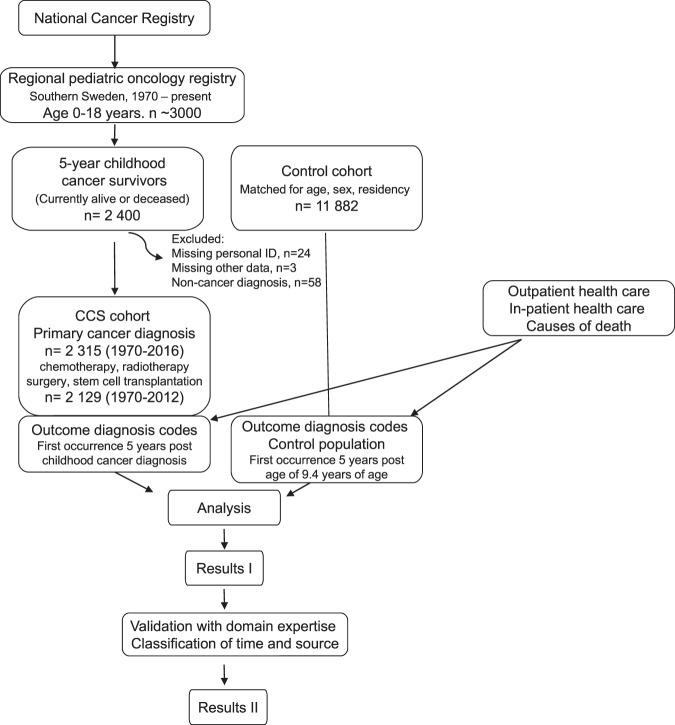
Outline of cohort study design and analysis. Recruitment was based on the presence in the national cancer registry and a regional registry, with a malignancy occurring between ages 0 and 18, between 1970 and 2016, currently living or deceased (*n* = 2315). Five controls were drawn from the general population and matched for year of birth, sex and place of residency (*n* = 11,882). For childhood cancer survivors (CCS), treatment data were gathered from the regional registry and assembled with outcome data from the national registries (outpatient, in-patient and cause of death). Outcomes were similarly assembled for the control group. Individuals missing personal identification numbers, missing other data and absence of a cancer diagnosis were excluded from the study. A total of 186 individuals contributed to the cohort with treatment variables but had not yet reached 5-year survival past date of diagnosis (1970–2012), and hence did not contribute with outcomes. After exclusions, the CCS cohort was 2129 in sample size. The sets were analyzed for sex-dependent health care usage, mortality and associations between treatments and outcomes as described.

This study assembled a population-based cohort of 5-year survivors and age-, sex-, and region-matched controls, as described in Fig. 1 and Supplementary Information “Data preparation Details”. The descriptive characteristics are shown in Table 1. The median time of follow-up was 15.1 years and the mean 16.4 years for in-patient and cause of death registry. For the outpatient care registry, the median time of follow-up was 13.3 years and the mean 11.8 years.

**Table 1:** Descriptive characteristics of the cohort and the control population.

	Control group	CCS
Total	11,882	2315
Alive	11,716 (98.6)	2145 (92.6)
Deceased	166 (1.4)	171 (7.4)
Male	5877 (49.5)	1152 (49.7)
Female	6005 (50.5)	1164 (50.3)
*Number of 5-year survivors per pediatric cancer diagnosis group*^81^		
Group		*n*, (%)
I	Leukemias	481 (20.8)
II	Lymphomas	292 (12.6)
III	CNS	561 (24.2)
IV	Neuroblastoma	87 (3.8)
V	Retinoblastoma	49 (2.1)
VI	Renal tumors	113 (4.9)
VII	Hepatic tumors	13 (0.6)
VIII	Malignant bone tumors	108 (4.7)
IX	Soft tissue sarcomas	95 (4.1)
X	Germ cell tumors	110 (4.7)
XI	Other malignant epithelial neoplasms and malignant melanomas	135 (5.8)
XII	Other malignant neoplasms	271 (11.7)
	Total	2315 (100)

The characteristics of the individuals contributing to the study are shown for the control group and childhood cancer survivors (CCS), respectively (total number and % of total). For the CCS group, the distribution of childhood cancer diagnoses is shown.

*n* number, *CCS* childhood cancer survivor, *CNS* central nervous system.

**Fig. 2:**
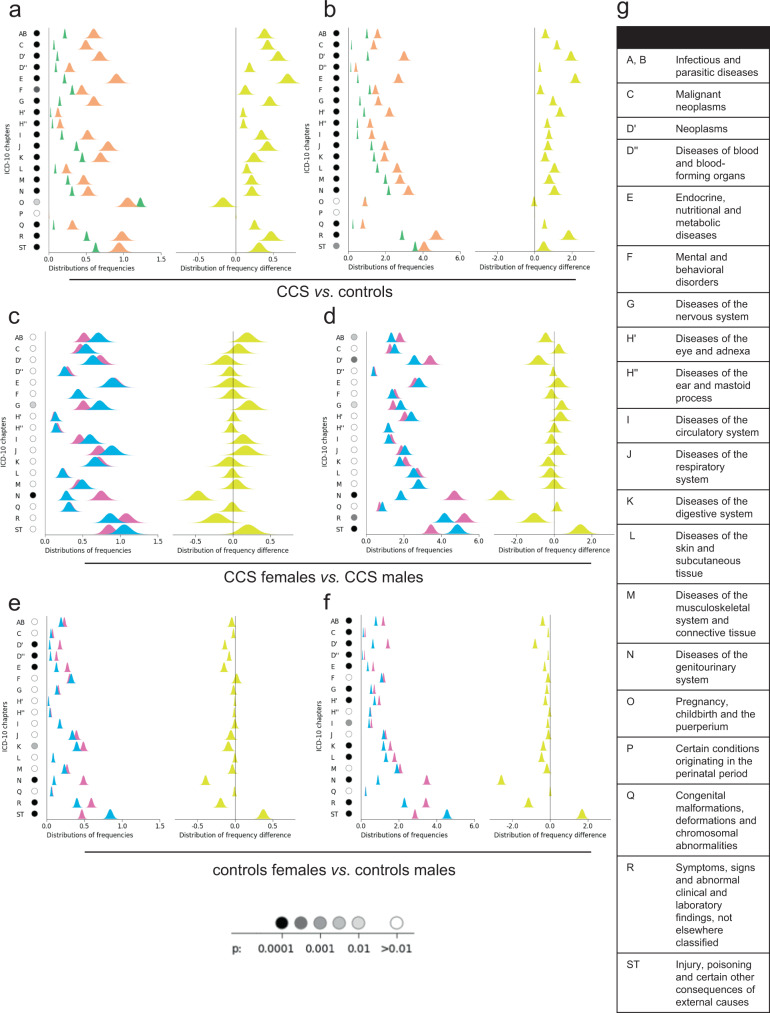
Distribution of frequency of outcomes for males and females, grouped by International Statistical Classification of Diseases and Related Health Problems 10th Revision (ICD-10) chapters. The left part of each panel shows the distributions of the frequency of occurrence in percent per year, according to Eq. 2 (childhood cancer survivors (CCS): orange bells; controls: green bells; males: blue bells; and females: pink bells). The right part of each panel shows the distribution of the difference between frequencies of the two compared groups in each diagram (as per Eq. 3). The sample size for the distribution of outcomes for CCS is *n* = 2129 independent samples (females: 1071, males: 1058). The control group was *n* = 11,882 (female: *n* = 6005, male: *n* = 5877). All distributions are normalized to unit height for ease of visualization. The significance is depicted by a gray scale in the circles to the left in each panel next to the ICD-10 chapter abbreviation. As the legend indicates, white is not significant and light gray to black is a logarithmic scale of significance levels from 0.01 to 0.0001. The results show that the incidence of outcomes is higher for the CCS than the control group for all ICD-10 chapters except pregnancy and childbirth (O) and perinatal conditions (P) (**a** in-patient setting; **b** outpatient setting). CCS males and females differed in health care usage for ICD-10 chapters G and N for in-patient care (**c**); and AB, D’, G, N, R, ST for outpatient care (**d**). CCS males sought more care than females both for in-patient and outpatient care with regard to neurological diseases (ICD-10 chapter G). The comparison for controls is similarly shown in **e** and **f** (in-patient care and outpatient care, respectively). For more details see the Results section. Full names of the ICD-10 chapters are shown in **g**. Source data can be found in Supplementary Data 1.

An initial comparison between the CCS cohort and the control population with regard to health care usage (Fig. 2), showed that the incidence of outcomes is higher for the CCS than the control group for all ICD-10 chapters except pregnancy and childbirth (O) and perinatal conditions (P) (Fig. 2a in-patient setting, and Fig. 2b outpatient setting). The uncertainty in the estimated frequencies, as seen by the more narrow distributions, is lower for the control group due to its larger size.

Next, we examined differences in health care usage between male and female CCS (Fig. 2c in-patient setting, and Fig. 2d outpatient setting). The results show that health care usage, differs significantly (*p* < 0.01) between males and females for ICD-10 chapters G and N for in-patient care; and AB, D’, G, N, R, ST for outpatient care (Fig. 2c, d, respectively). These results are mirrored in the control group where significant differences between males and females were found in ICD-10 chapters AB, C, D’, D”, E, G, H, I, K, L, N, R, and ST for outpatient care (Fig. 2f). For in-patient care, control group males and females differed significantly in chapters D’, D”, E, K, N, R, ST (Fig. 2e).

When examining only significant results, the results show that females (both CCS and controls) sought health care more often than males, except for outcomes from ICD chapter ST (Injury, poisoning and certain other consequences of external causes) where males (both CCS and controls) dominated. The only statistically significant exception to this pattern was for neurological diseases (ICD-10 chapter G), where CCS males sought more care than females both in the in-patient and outpatient setting. Again, the larger size of the control population resulted in higher significances of the differences (visualized as more narrow distributions in Fig. 2) within the control group (Fig. 2e, f), than within the CCS group (Fig. 2c, d) even if the magnitudes of the differences in most chapters are similar. Source data can be found in Supplementary Data 1.

**Fig. 3:**
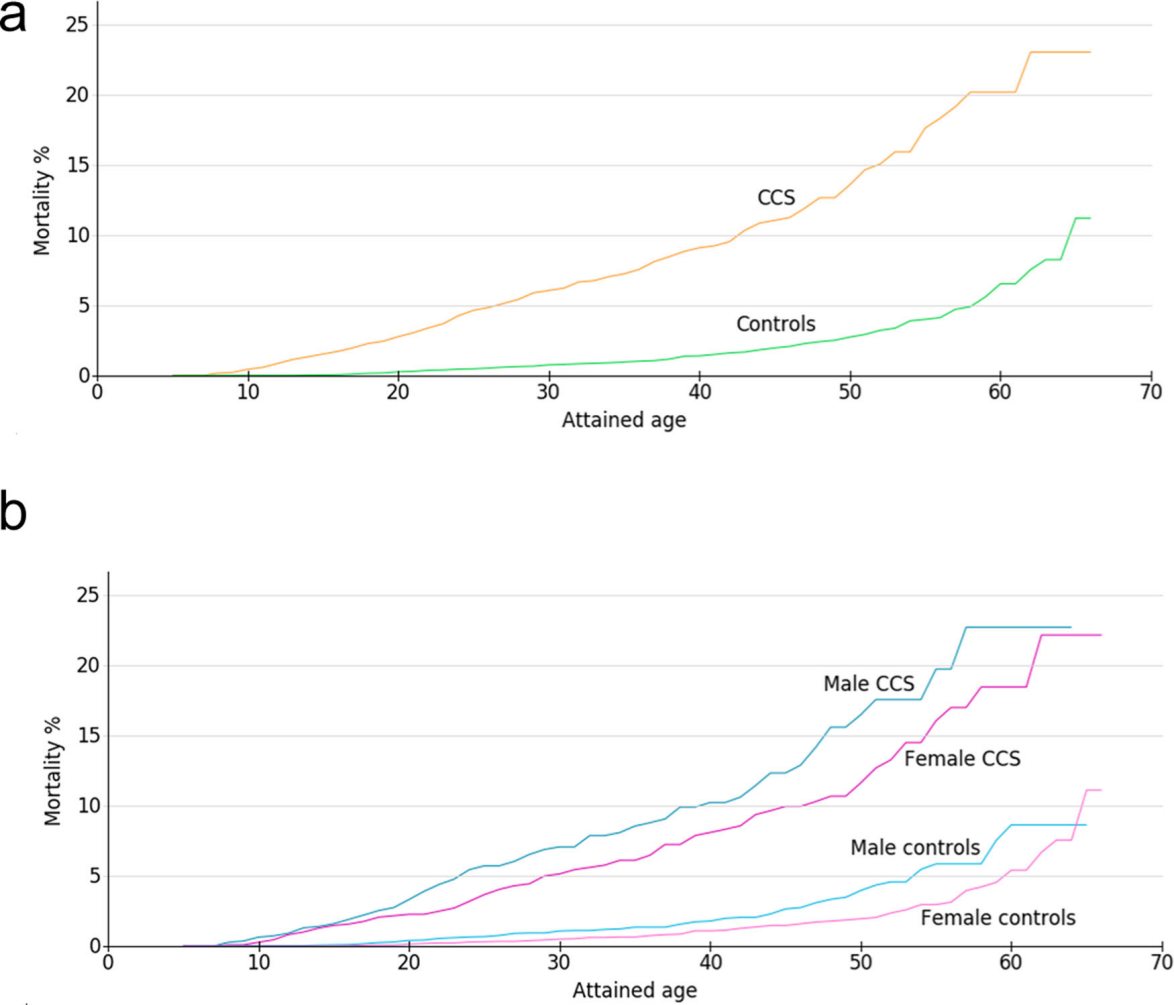
Mortality is higher in childhood cancer survivors (CCS) than in controls. Mortality of (**a**) CCS (orange line) as calculated by Kaplan–Meier and compared to the control group (green line) and divided by sex (**b**) (males: blue line; female: pink line) depicted by attained age. The sample size for calculation of mortality was for CCS is *n* = 2315 biologically independent samples (CCS female: *n* = 1163, male: *n* = 1152). The control group was *n* = 11,882 (female *n* = 6005, male *n* = 5877). There were 171 deaths in the CCS group and 166 deaths in the control group. Source data can be found in Supplementary Data 2.

For descriptive purposes, the mortality rate was determined for CCS and controls (Fig. 3a), and males and females were compared (Fig. 3b; male blue line, female pink line). The CCS cohort showed an approximately five times higher death rate at each attained age compared to controls. For CCS as well as controls, mortality was higher in males than females. Source data can be found in Supplementary Data 2.

A substantial result of this study was the developed method for causal inference from limited size data sets. The analysis approach was to use a constraint-based method for causal inference, the PC algorithm^11,12^, to build a causal graph between all individual properties, childhood cancer diagnoses, cancer treatments, and potential late effects. However, methods for causal inference are typically demanding in terms of the amount of data required. Constraint-based methods rely on a conditional independence test to first build a graph of all potential causal relations and then successively remove links that can be explained by causation via other variables. This may require conditioning each test on several variables, and each such conditioning will reduce the effective sample size for the test, reducing the significance of the test. The limited amount of medical data, especially after subdivision into treatment groups, in the current study would hence pose a serious threat to this approach. We solved this by developing a specially tailored conditional independence test, based on Bayesian estimation of distributions and a measure of correlation that is invariant under conditioning. This makes it possible to base the estimation of the correlation on the whole data set also when conditioning on one or more variables, and thus makes it possible to maintain a high level of significance if the effect is strong enough, despite a limited amount of data. However, when simultaneously controlling for a large number of variables this approach will also ultimately result in loss of statistical power. In specific, when each partition only contains samples from one of the compared groups, i.e., there is no information left in the partitions, then not even pooling over the partitions will counteract the loss of significance. Fortunately, the PC algorithm demonstrates efficiency in selecting the smallest subsets of variables that need controlling for, and no capping of the number of simultaneously controlled variables was necessary for this study.

The analysis identified 98 significant associations between childhood cancer diagnoses, treatment details and outcomes with an estimated number of false-positive results of approximately 1.6% (*n* = 2). The associations between treatment and well-known late effects (Category A) accounted for 74.5%; 5.1% were suggested as novel effects (Category B; shown in Table 2), and 20% (Category C) represented other findings (all data shown in Supplementary Data 3 and 4).

**Table 2:** The presumed novel associations (*p* < 0.0001) between childhood cancer diagnosis (CCD), treatment details and outcomes (shown in plain text and International Statistical Classification of Diseases and Related Health Problems 10th Revision (ICD-10) codes).

Association number as found in Supplementary Data 3	Presumed novel associations (*n* = 5)		Literature references
	Possible cause	Outcome	
2	Alkylating agents	Disorders of lacrimal system (H04)	^70–72^
7	Topoisomerase inhibitors	Viral infection of unspecified site (B34)	n.a.
49	Surgery in pituitary region	Viral intestinal infection (A08)	n.a.
50		Gastroenteritis and colitis of infectious origin (A09)	n.a.
72	Malignant neoplasm of cervix uteri (C53)	Inflammatory disease of uterus, except cervix (N71 | female)	n.a.

Analysis was performed with the developed method as described and validated with domain expertise. The sample size was *n* = 2315. These five associations were graded B as presumed novel in the validation step with domain expertise and literature searches. They were regarded as intact as they did not originate from the outpatient setting, were not present before the CCD, and did not occur within the 0.5–5 years post CCD time frame. The 98 associations are shown in full in Supplementary Data 3 and the statistical basis for them is shown in Supplementary Data 4.

|Female or |male indicates that the association was based on only one sex, respectively.

International Statistical Classification of Diseases and Related Health Problems 10th Revision (ICD-10) codes in parentheses.

*n.a.* not applicable as none were found.

Three lines of unexpected findings led to further questioning. Firstly, a surprisingly high number of consequences were associated with use of topoisomerase inhibitors. We hypothesized that the entry of this chemotherapeutic class into treatment protocols in the 1990s influenced the results and this led us to control for time periods as a confounding factor. The results show that four of the five associations concerning the use of topisomerase inhibitors lost their status as true associations, as did testicular dysfunction as a consequence of vinca-alkaloid use (Supplementary Data 3, associations 4, 8–11, marked C4) after controlling for time with a cut-off at the year 1988.

Secondly, an unexpected number of benign or malignant neoplasms (*n* = 17) were found as consequences of pediatric malignancy. As second malignant neoplasms are well-known late effects after childhood cancer, this finding warranted additional attention. Therefore, all associations containing ICD-10 chapter C or chapter D outcome codes were examined in depth. We found that 5 of the 17 associations were accounted for as originating from the outpatient setting only. An additional two associations where ICD-10 chapter C or D outcome codes were effects of other causes, were also found to stem from the outpatient setting. These were malignancy of the skin following radiation to the brain, and malignant neoplasm of spinal cord, cranial nerves and other parts of central nervous system following surgery to the pituitary region. This line of questioning is discussed below.

Thirdly, we suspected that some outcome codes were routinely repeated by health care although the disease episode represented a historic medical event. Therefore, we determined the time point of outcome code registration for all 98 associations, more specifically if it was present exclusively 5 years after the CCD. The results show that 9.2% of the total number of outcome codes originated prior to the CCD, and that 10.2% originated in the 0.5–5-year period after CCD. We could thereby account for 15 of the 21 associations, which had been categorized as other findings (Category C). Six associations of the category Other findings remained intact, i.e., significant associations which clinical expertise could not corroborate nor reject under the current circumstances. When examining the 17 neoplasms that were identified as consequences of a pediatric malignancy, after this third step, seven associations with the outcomes benign or malignant neoplasms, remained intact.

After controlling for time periods, and accounting for time point of outcome code registration and source of the outcome codes, 66 of the 98 (67.3%) associations remained intact, i.e., presented as consequences of childhood cancer or its treatment occurring solely 5 years post CCD at a maintained level of significance. The chemotherapeutics classes alkylating agents, anthracyclines, and topoisomerase inhibitors were identified as causes of six effects. Distinct radiation fields were identified as causes of 31 effects. Surgeries accounted for six effects, and stem cell transplantation accounted for three. Preexisting conditions accounted for one effect. There were 16 consequences of pediatric malignancies.

**Fig. 4:**
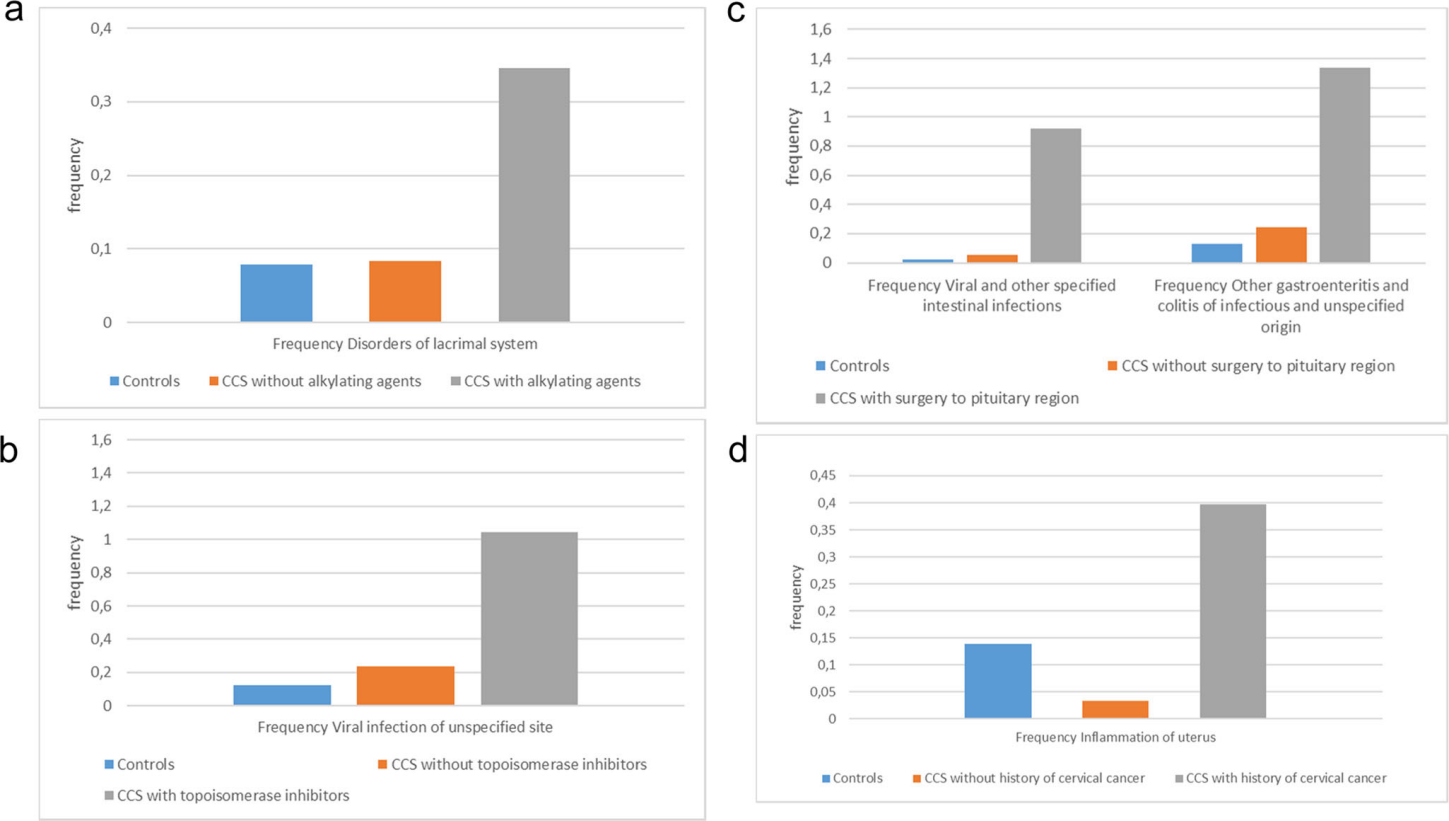
The frequency of the five presumed novel associations between treatments and outcomes. The frequency of the novel associations was calculated by dividing number of individuals by the accrued number of person-years for the control population (blue bars), childhood cancer survivors (CCS) without the specific childhood cancer diagnosis (CCD) or treatment history (orange bars) or CCS with the specific CCD or treatment history (gray bars). The sample sizes for frequency analysis were as follows: **a** outcome lacrimal disorders: with alkylating agents, *n* = 768, without alkylating agents *n* = 1547 and controls *n* = 11,882. **b** Outcome unspecified virus infection: with topoisomerase inhibitors *n* = 408, without topoisomerase inhibitors: *n* = 1907, controls: *n* = 11,882. **c** Left: outcome: viral and other specified intestinal infections: with pituitary surgery: *n* = 61, without pituitary surgery: *n* = 2254, controls: *n* = 11,882. **c** Right: outcome: other gastroenteritis and colitis of infectious and unspecified origin. Sample size is identical to panel **c**, left. **d** Outcome: endometritis: female CCS with C53 (malignancy of the cervix): *n* = 140, female CCS without C53: *n* = 1023, female controls: *n* = 6005. Source data can be found in Supplementary Data 4.

The five presumed novel associations, Category B in Table 2, are: alkylating agents as cause of the lacrimal system disorders; mostly dry eyes and stenosis of lacrimal ducts (association 2, controlled for time with a cut-off at the year 1988), topoisomerase inhibitors as cause of unspecific viral infection, (association 7, controlled for time with a cut-off at the year 1988), surgery in the pituitary region as a cause of viral intestinal infection and gastroenteritis, and colitis of infectious origin (association 49–50, controlled for time with a cut-off at the year 1996), and “inflammatory disease of uterus, except cervix” as a consequence of malignant neoplasm of cervix uteri (association 72, controlled for time with a cut-off at the year 1996). The frequency of the previously unreported associations identified in this study in CCS with vs without that specific CCD or treatment history, and the control population is shown in Fig. 4a–d. Source data can be found in Supplementary Data 4.

## Discussion

The results for this newly assembled cohort of 5-year survivors of childhood cancer in southern Sweden confirm that CCS seek health care to a higher extent than the general population^13,15–18^. The patterns for outcomes regarding males and females were similar between CCS and the general population, except for causes related to the nervous system, for which females in the general population sought more health care, whereas the males among the CCS sought more health care. Previous studies confirm conditions related to the nervous system being a large cause for hospitalization among CCS^17^. The overall mortality of the CCS cohort was, as expected, higher than for the control population^19^, with males showing higher mortality than females among both CCS and control population.

The validation of the causal analysis showed that the majority of the identified associations were well known and have been described^20–68^ previously, as shown in Supplementary Data 3. These include cardiomyopathies following anthracycline treatment, endocrine disorders by multiple causes, e.g., radiation, surgery and chemotherapy, and serious medical events following pediatric brain tumor. The results show, as expected, that radiotherapy to the brain caused a larger number of reasons for seeking medical attention later in life than any other treatment (associations 21–32, Supplementary Data 3) and these remained intact after investigation of time of the outcome code. However, several well-known late effects, e.g., secondary cancers, cognitive effects or diabetes were not identified. This may be due to variation in coding for the medical event, or that the number of cases was too few to reach the stringent level of significance selected for this study. With specific regard to neurocognitive late effects, such impairments may be subtle and diagnostically undetected resulting in them being under-reported.

The statistical model used in the causal analysis assumes exponentially distributed events. This assumption means that the risk of a late effect is independent of the time since treatment, and of the age of the subject. Other distributions, such as Gamma or Weibull, could more realistically catch time dependency of the risk of a late effect. Such distributions could have been used instead but would require another invariant expression of the independence test. The concept of “added risk” is simple and intuitive, and the key enabler of the technique when the amount of data is limited. We expect the detrimental effect of making the exponential approximation to be small. Even though the risk may actually vary with time for an individual, on a population basis consisting of a heterogeneous mix of individuals of different ages and observation times, these differences would cancel each other out and the “average” added risk would be detected by the current method. There is a possibility that some weaker effect is not detected because of the approximation, but on the other hand, a more elaborate model with more parameters would require more data, and would therefore also risk failing at identifying such weak effects (the “bias-variance” tradeoff).

The invariance assumption that is also used essentially means that the effect of different treatments can be considered separately. If there are synergy effects, the magnitude of the synergy effect will be misjudged by the method. If so, the method will detect the “average” effect of factors separately. Furthermore, some factors may have a multiplicative effect rather than additive, such as a factor controlling how sensitive the person becomes to other factors. One obvious example of this is sex, which affects how common or even possible some outcomes are. Specifically for sex, this is handled by performing the analysis separately for males and females for the outcomes that are dependent on sex (e.g., intact associations 3, 15, 18, 39, etc. in Supplementary Data 3). The level of detail present in the treatment history in the assembled cohort is a strength that allows us to link particular treatment details to specific outcomes. As treatment regimens for childhood cancer are multimodal and may include several agents administered together as part of a protocol, it is a challenge for the research field, as well as for our study, to deem one single factor responsible for a particular outcome. Others report on approaching such challenges through other mathematical models^69^. The succession of treatment protocols for childhood cancer signals caution for time as a confounding factor, as seen in the current study (Supplementary Data 3, associations marked with C4). Controlling for time as a confounder is in the general case rather complicated, as we cannot rely on Gaussian distributions or linear trends. Ideally, we would partition the time axis into a large number of sections, each to be considered separately. Such partitioning would dramatically decrease the number of samples per partition and have a negative impact on the statistical power. Therefore, this was not done across all associations in the current study. The approach that was used instead relied on domain expertise and manual investigations. If a representative time point can be identified for when a treatment regime or diagnosis coding convention changed, it is sufficient to split the time axis in two parts at that specific time point and then controlling associations involving that specific factor. This approach limited loss of statistical power and was therefore feasible also for the limited amount of data in this study. The associations identified as presumably novel in this study (Table 2) were controlled for time in this way to ensure their validity.

Iterative analyses in collaboration with generalist and specialist competencies have played a vital role throughout this study. In two cases (associations 6 and 67, Supplementary Data 3) the domain experts could refute the method, namely when anthracyclines were identified as the cause of scarring of skin, but further investigation showed that the association was most likely due to placement of central venous catheter for administration of all/any chemotherapy agents. Also, the method did not identify an association between cisplatin and hearing loss, a well-established late effect and its cause.

Our study found five presumed novel associations, which we discuss in the following: we report on alkylating agents as the cause of disorders of the lacrimal system; mostly dry eyes and stenosis of lacrimal ducts. In support of this finding, we identified one retrospective study including 128 adult women in early-stage breast cancer treatment that reported transient ocular toxicity, e.g., epiphora in 18% of the cases in close conjunction to therapy which included cyclophosphamide, methotrexate and 5-fluorouracil^70^. A publication from 1979 first reported the alkylating agent cyclophosphamide as causing transient blurred vision (disappeared 1 h to 14 days post administration) in five children with cancer^71^. Cyclophosphamide has been reported to cause *keratoconjunctivitis sicca* and blepharoconjunctivitis^72^. It is surprising that the lacrimal system was impaired 5 years after CCD, long after treatment cessation.

We found unspecific viral infection as a supposed consequence of topoisomerase inhibitor use. CCS are more prone to infections than the general population but to our knowledge, this is the first report where specifically topoisomerase inhibitors are associated with this effect.

One of the findings concerned female reproductive health: endometritis, identified as an outcome after cervical cancer. Endometritis, an inflammation of the uterine lining is treated with antibiotics if the origin is infectious. Chronic endometritis alters the cellular and immunological balance of the uterine mucosa, and appears to impair implantation as treatment with antibiotics improved the rate of live births in affected women^73^. To what extent endometritis contributes to infertility in the CCS population is undetermined, and is most likely overshadowed by other fertility-impairing agents or treatment modalities. The majority of adult cases of cervical cancer are attributed to human papilloma virus infection. We cannot account for the etiology of cervical cancer in the cohort although others report on examples of hereditary patterns and iatrogenic causes^74,75^. Additional studies would provide details to create a more complete representation of this association.

The finding of gastrointestinal infections occurring 5 years post CCD, as a consequence of surgery in the pituitary region, is a surprising finding. The reason for this entry into the outcomes registries could be increased surveillance after hypophysectomy and quicker medical action to a CCS presenting with a gastrointestinal infection than an otherwise healthy individual would receive. Yet, a Nordic study of long-term outcomes of CCS (*n* = 21,297), reported a 1.9-fold change compared to the expected, for hospitalization due to gastrointestinal infections^17^, but without suggesting a cause. Disruptions to the brain–gut axis cause changes in responses of the nervous system and may lead to GI disorders^76^. It is established that pediatric patients diagnosed with pituitary malignancies (e.g., craniopharyngioma, which account for >80% of these), and undergo surgery to the pituitary region, later develop metabolic syndrome due to disturbances in the endocrine axis. Unsurprisingly, tumor localization within this region, and hence the surgery used to remove it, influences the outcome in terms of obesity^28,77^. Animal studies showed that hypophysectomy caused atrophy of the intestinal mucosa and increased susceptibility to bacterial, viral and nematode infections^78^ concomitant with reduced serum IgG and IgM and intestinal IgA^79^. This demonstrates that pituitary hormones are vital for maintaining the integrity of the intestine, and that the systemic and intestinal humoral immune responses are affected by surgery in the pituitary region, in support of our findings. Future studies may address whether pituitary lesions, or surgery thereof play a driving role in the development of the metabolic syndrome in other populations than CCS.

Factors that influence the likelihood of success of registry studies are (1) the presence or absence of recordings, (2) a skew for financial benefit, and (3) the influence of the physician or clinical coding culture. Our grading suggests that >5% of the associations were a consequence of diverse coding cultures or choices (Supplementary Data 3, category C1). We speculate that ~10% of the results (Supplementary Data 3, category C2) are a result of increased surveillance for, and awareness of SMN that may have resulted in benign neoplasms being found. Late effects after childhood cancer are generally assumed to stem from the therapy given at a young age rather than the primary malignancy itself, although this is under questioning. A surprisingly high number of benign and malignant consequences of pediatric malignancies were initially identified by our analysis. We were, due to the unobtainability of medical charts, not able to distinguish between actual clinical outcomes and registry-based biases and therefore questioned the reliability of these results. If a CCD can be treated with more than one treatment strategy, and all treatment modalities contribute to the outcome, then the method will point to the primary diagnosis as the (strongest) cause. Furthermore, to address the possibility of registration biases, we performed an examination to pinpoint source and timing of coding of outcomes. In the case of coding originating from the outpatient setting, this could result from either a routine (and sometimes imprecise representation) repetition of the in-patient setting code from the past (assumed in Supplementary Data 3, association 51), or an actual surveillance performed in the outpatient setting (assumed in Supplementary Data 3, associations 21 and 70). A subset of the outcome codes identified in the 98 associations was present prior to the childhood cancer diagnoses and others were, in addition to later than 5 years post CCD, also present in the 0.5–5-year period post CCD. Hence, our question of whether routine repetition caused clouding of the results was in part answered. We cannot confirm solely routine repetition, and instead, this raises the question of when late effects emerge in relation to the conventional 5-year post CCD date. Our results imply that late effects emerge sooner and if extrapolated, that approximately 10% may remain undetected if strictly keeping to the 5-year mark in registry studies. Although the outpatient and in-patient registries have different starting dates, the results do not suggest a lead time bias that would compete with the more human-factor-oriented uncertainties associated with especially the outpatient registry. The rationale behind the inclusion of outcome codes from in-patient and outpatient care was to be inclusive of all degrees of medical severity, and pursue a hypothesis-generating approach with a new method applied to a developing field. Health issues that are not life-threatening can still affect survivors’ quality of life. Indeed, the five associations identified as a presumably novel point to mild and treatable conditions. Whether the findings will influence medical follow-up is uncertain. Instead, the findings will more likely contribute to a broader understanding of the consequences of cancer treatment.

The current study draws conclusions from a small, yet detailed, population-based cohort which was iteratively analyzed with generalist and specialist competencies and appropriate methods. Bayesian statistics have the advantage of enabling full use of the information available in the data, while keeping control of the uncertainty. Bayesian statistics have shown, and with this study continue to show, potential in medical studies as an important complement to classical statistical methods. The findings presented here may shed new light on biological causes of late effects, and future studies in larger cohorts, or of in vitro character may corroborate the findings. Furthermore, the presented method may lend itself to other uses where data are scarce.

## Supplementary information

Supplementary Information

Supplementary Data 1

Supplementary Data 2

Supplementary Data 3

Supplementary Data 4

Description of Additional Supplementary Files

Reporting Summary

## Data Availability

Restrictions apply to the availability of the underlying data set, which was used under specific conditions for the current study. The reason for not making the data set publicly available is that the rareness of the disease and the distinct geographical catchment area could place the anonymity of the study subjects at risk. Data are however available from the authors upon reasonable request and with permission of the ethical review board. Source data can be accessed as Supplementary Data files 1–4.

## Code availability

All custom codes are deposited in GitHub (https://github.com/Anders-Holst/Bonsai)^80^.
