# Supplementary material for: Identifying causal relationships of cancer treatment and long-term health effects among 5-year survivors of childhood cancer in Southern Sweden
Source: Commun Med (Lond). 2022 Mar 2;2:21. doi: 10.1038/s43856-022-00081-z (PMC9053221; doi:10.1038/s43856-022-00081-z)
Supplement: Supplementary file 1 — Supplementary Information [file 43856_2022_81_MOESM1_ESM.pdf]

Supplemental Information file

Identifying causal relationships of cancer treatment and long-term health effects among 5-year survivors of childhood cancer in Southern Sweden

Anders Holst [1], Jan Ekman [1], Magnus Ahrholt-Petersson [2], Thomas Relander [3], Thomas Wiebe [4], Helena M. Linge *[4]

Affiliations: [1] Department of Computer Science, Division of Digital Systems, Research Institutes of Sweden, Stockholm, Sweden, [2] Climber, Malmo, Sweden, [3] Department of Clinical Sciences Lund, Oncology, Skane University Hospital, Lund University, Lund, Sweden, [4] Department of Clinical Sciences Lund, Pediatrics, Faculty of Medicine, Lund University, Lund, Sweden.

Supplementary Methods

Data preparation details

The schematic outline of the study is shown in figure 1. The demographical data and detailed treatment data of all 5 year survivors (n=2400; living and deceased, above and below 18 years of age) in the regional quality registry BORISS [1] was extracted. The basis of their inclusion in this registry was the registration in the national cancer registry. For each childhood cancer survivor, five control subjects from the general population were selected (or fewer if not 5 matches could be found) based on sex, year of birth and place of residency, and included in the study (n= 11 882). The individuals drawn for the control population only occur once in the cohort and could only be used to match one single CCS. For both survivors and controls the respective outcomes data was extracted and aligned by the Statistical Services at the Swedish National Health and Welfare Board. The datasets were pseudonymized and then returned for analysis. In this step 58 CCS were excluded due to lack of cancer diagnosis codes (as confirmed by pathology reports), 24 CCS due to lack of valid personal ID numbers, and 3 CCS due to missing other essential registry data, resulting in 2315 remaining CCS cases. They were diagnosed with a childhood cancer between the years 1970 and 2012. The patients diagnosed with childhood cancer in 2013-2015 were not yet 5-year survivors (n=186) at the time of data extraction. The detailed treatment data from these patients contributed to the analyses but their outcomes did not.

For this study the outcomes data included in-patient care, out-patient care, and causes of death. All ICD-7, 8 and -9 codes among the outcome codes were converted to ICD-10 by two of the authors and cross-checked. We used guides issued by the Swedish National Health and Welfare Board and on-line resources (icd.internetmedicin.se; https://icd.who.int/). The chapters are as follows: A,B: Certain infectious and parasitic diseases; C, D': Neoplasms; D': Diseases of the blood and blood-forming organs and certain disorders involving the immune mechanism; E: Endocrine, nutritional and metabolic diseases; F: Mental and behavioral disorders; G: Diseases of the nervous system; H': Diseases of the eye and adnexa; H'': Diseases of the ear and mastoid process; I: Diseases of the circulatory system; J:

Diseases of the respiratory system; K: Diseases of the digestive system; L: Diseases of the skin and subcutaneous tissue; M: Diseases of the musculoskeletal system and connective tissue; N: Diseases of the genitourinary system; O: Pregnancy, childbirth and the puerperium; P: Certain conditions originating in the perinatal period; Q: Congenital malformations, deformations and chromosomal abnormalities; R: Symptoms, signs and abnormal clinical and laboratory findings, not elsewhere classified; ST: Injury, poisoning and certain other consequences of external causes. Chapters U, V, and Z were excluded from analysis due to lack of relevance.

The coding structure for chemotherapeutic agents (n=90), anatomical site of radiation therapy (n=145), and stem cell transplantation (allogeneic, autologous) was in place upon extraction from the BORISS registry [1].

The chemotherapeutic agents were grouped into 10 classes as follows: A= alkylating agents (n=20), B= antimetabolites (n=12 //15 if taking into account route of administration//), C= anthracyclines (n=5), D= vinca alkaloids (n=4), E= inhibitors of tyrosine kinases (n=7), F= steroids (n=5), G= antibody (n=5), H= topoisomerase inhibitors (n=5), I = other cytostatic effects (n=15) and K= modulating agents (n=9). The field codes for anatomical sites of radiation therapy were congregated into the fields: face (n=11), abdomen (n=35), extremity (n=20), neck (n=16), brain (n=21), spinal canal (n=5), major fields (n=2, Total Marrow Irradiation and Total Body Irradiation), testicles (n=2), thorax (n=29), and eye (n=4).

The surgical procedures were found in plain text in the registry and were for this study coded according to the Swedish version of NOMESCO Classification of surgical procedures v 1.9 [2] with two letters. In order to distinguish between the anatomical sites of CNS surgery, which is of particular importance in treatment and follow-up of pediatric malignancies, we introduced five additional levels of coding under AA: Cranial and intracranial structures: AA1: cerebrum, AA2: cerebellum, AA3: pituitary gland

including sella turcica, craniopharyngeom, and suprasellar position, AA4: Pineal gland, and AA5: brainstem.

The predisposing medical conditions or syndromes in the CCS cohort which were considered in the analysis were (abbreviations and alternative nomenclature in parenthesis): malignant endocrine neoplasia type 2 (MEN2, Sipples, Sippels), neurofibromatosis (NF1, NF-1, Recklinghausen's syndrome, Recklinghausen, Mb Recklinghausen), Down syndrome (MbDown, morbus Down, Trisomy21, Trisomy-21, trisomy21). We excluded any outcome code that was identical to the first CCD if it occurred later than the 5-year-since diagnosis date based on the assumption that it was a routine repetition.

To compare CCS health care usage with the control population we introduced a mock time-of-diagnosis as the starting point for the comparisons. The average age of diagnosis among CCS was 9,4 years. The same age was used as a starting point for the control population. The observation time was from date of diagnosis (or 9,4 years of age for the control group) to a) The first registered event, b) date of death, c) possible emigration of the patient, d) the end date of the registers. In the last two cases the observation was censored one-sidedly. For outpatient care, starting in the year 1997, a 2-sided censoring was applied.

Choice of significance level

In the current study, we did not test a single hypothesis, but searched broadly for correlations. This requires a careful consideration of significance levels. If a significance level of 5% were selected, this would mean that 5% of all performed tests would indicate a significance correlation when in fact there was none. If all performed hypotheses tests were independent, we could have divided the final desired significance level by the number of tests. However, treatments and childhood cancer diagnoses are all correlated, as are the different outcomes, which warrants the approach of causal inference on the whole graph. The alternative is to use a Bayesian approach, and select a significance level which will make the probability that a found correlation is spurious acceptably small. A threshold for this probability can be achieved by considering the number of found correlations at different significance levels.

In this study we found 274 potential causal relations at the 0.01 significance level. Of those, 146 were at a significance level in the interval 0.001 – 0.01, 32 at a significance level in the interval 0.0001 – 0.001, and 94 at a significance level in the interval 0.0 – 0.0001. If we, as a worst case assume that all 146 found relations with significance level in the interval 0.001 – 0.01 are in fact spurious, then we would expect a tenth this many spurious cases in the ten times smaller interval 0.0001 – 0.001, that is some 15 out of 32 found relations, and still 9 times fewer in the interval 0.0 – 0.0001, that is around 1.6 out of 98 relations. This is less than 2%, which was considered small enough. We therefore continued the analysis with the 98 relations that had a significance level better than 0.0001 (keeping in mind that around 2 of them may be spurious).

Supplementary References

1. Wiebe, T., Hjorth, L., Marotta Kelly, M., Linge, H. M. & Garwicz, S. A population based pediatric oncology registry in Southern Sweden: the BORISS registry. *Eur. J. Epidemiol.* **33**, 1125-1129 (2018).

2. *Swedish version of NOMESCO Classification of surgical procedures* (eds Ortendahl, C. & Rosen, M.) (NOMESCO and Socialstyrelsen (Swedish National Health and Welfare Board), Lindesberg Sweden, 1997 revised 2004).
