# Supplementary material for: Identifying causal relationships of cancer treatment and long-term health effects among 5-year survivors of childhood cancer in Southern Sweden
Source: Commun Med (Lond). 2022 Mar 2;2:21. doi: 10.1038/s43856-022-00081-z (PMC9053221; doi:10.1038/s43856-022-00081-z)
Supplement: Supplementary file 6 — Description of Additional Supplementary Files [file 43856_2022_81_MOESM6_ESM.pdf]

Description of Additional Supplementary Files

File Name: Supplementary Data 1

Description: This is supplemental data 1 which contains source data to Figure 2.

File Name: Supplementary Data 2

Description: This is supplemental data 2 which contains source data to Figure 3.

File Name: Supplementary Data 3

Description: This is supplemental data 3 which contains all associations identified in the study.

File Name: Supplementary Data 4

Description: This is supplemental data 4 which contains source data to Figure 4, Table 2 and Supplementary Data 3.
