# Supplementary material for: Identifying causal relationships of cancer treatment and long-term health effects among 5-year survivors of childhood cancer in Southern Sweden
Source: Commun Med (Lond). 2022 Mar 2;2:21. doi: 10.1038/s43856-022-00081-z (PMC9053221; doi:10.1038/s43856-022-00081-z)
Supplement: Supplementary file 7 — Reporting Summary [file 43856_2022_81_MOESM7_ESM.pdf]

nature research

Corresponding author(s): Helena Linge

Last updated by author(s): Jan 26, 2022

Reporting Summary

Nature Research wishes to improve the reproducibility of the work that we publish. This form provides structure for consistency and transparency in reporting. For further information on Nature Research policies, see our Editorial Policies and the Editorial Policy Checklist.

Statistics

For all statistical analyses, confirm that the following items are present in the figure legend, table legend, main text, or Methods section.

n/a	Confirmed	
☐	☒	The exact sample size (*n*) for each experimental group/condition, given as a discrete number and unit of measurement
☐	☒	A statement on whether measurements were taken from distinct samples or whether the same sample was measured repeatedly
☐	☒	The statistical test(s) used AND whether they are one- or two-sided<br>*Only common tests should be described solely by name; describe more complex techniques in the Methods section.*
☐	☒	A description of all covariates tested
☐	☒	A description of any assumptions or corrections, such as tests of normality and adjustment for multiple comparisons
☐	☒	A full description of the statistical parameters including central tendency (e.g. means) or other basic estimates (e.g. regression coefficient) AND variation (e.g. standard deviation) or associated estimates of uncertainty (e.g. confidence intervals)
☐	☒	For null hypothesis testing, the test statistic (e.g. *F*, *t*, *r*) with confidence intervals, effect sizes, degrees of freedom and *P* value noted<br>*Give P values as exact values whenever suitable.*
☐	☒	For Bayesian analysis, information on the choice of priors and Markov chain Monte Carlo settings
☒	☐	For hierarchical and complex designs, identification of the appropriate level for tests and full reporting of outcomes
☐	☒	Estimates of effect sizes (e.g. Cohen's *d*, Pearson's *r*), indicating how they were calculated

Our web collection on statistics for biologists contains articles on many of the points above.

Software and code

Policy information about availability of computer code

Data collection	The data was collected from The Council of Skåne and from the National Health and Welfare board respectively, after approval from the ethical review board (permit number 2018-022)
Data analysis	Custom code was used for the analyses. All custom code is deposited in GitHub (https://github.com/Anders-Holst/Bonsai)

For manuscripts utilizing custom algorithms or software that are central to the research but not yet described in published literature, software must be made available to editors and reviewers. We strongly encourage code deposition in a community repository (e.g. GitHub). See the Nature Research guidelines for submitting code & software for further information.

Data

Policy information about availability of data

All manuscripts must include a data availability statement. This statement should provide the following information, where applicable:

- Accession codes, unique identifiers, or web links for publicly available datasets
- A list of figures that have associated raw data
- A description of any restrictions on data availability

Restrictions apply to the availability of the underlying dataset, which was used under specific conditions for the current study. The reason for not making the dataset publicly available is that the rareness of the disease and the distinct geographical catchment area could place the anonymity of the study subjects at risk. Data are however available from the authors upon reasonable request and with permission of the ethical review board. Source data can be accessed as Supplementary Data files 1-4.

Field-specific reporting

Please select the one below that is the best fit for your research. If you are not sure, read the appropriate sections before making your selection.

☒ Life sciences ☐ Behavioural & social sciences ☐ Ecological, evolutionary & environmental sciences

For a reference copy of the document with all sections, see nature.com/documents/nr-reporting-summary-flat.pdf

Life sciences study design

All studies must disclose on these points even when the disclosure is negative.

Sample size	The sample size is based on a population based registry. The smaller size of the cohort (compared to other cohorts) warranted the development of the method which is described in the manuscript.
Data exclusions	58 CCS were excluded due to lack of cancer diagnosis codes (as confirmed by pathology reports), 24 CCS due to lack of valid personal ID numbers, and 3 CCS due to missing other essential register data, resulting in 2315 remaining CCS cases. 186 cases were included based on treatment data but as they were not yet 5-year survivors their outomes did not contribute to the analysis.
Replication	The analysis yielded the same results upon replication but, we cannot answer towards reproducibility as we did not study more than one dataset. Also, other data sets have different variables.
Randomization	The study paired treatment data with outcome data and significant associations were identified. No randomization was used.
Blinding	Blinding was not in place as we used the opportunity for cross disciplinary learning.

Reporting for specific materials, systems and methods

We require information from authors about some types of materials, experimental systems and methods used in many studies. Here, indicate whether each material, system or method listed is relevant to your study. If you are not sure if a list item applies to your research, read the appropriate section before selecting a response.

Materials & experimental systems

n/a	Involved in the study	
☒	☐	Antibodies
☒	☐	Eukaryotic cell lines
☒	☐	Palaeontology and archaeology
☒	☐	Animals and other organisms
☐	☒	Human research participants
☐	☒	Clinical data
☒	☐	Dual use research of concern

Methods

n/a	Involved in the study	
☒	☐	ChIP-seq
☒	☐	Flow cytometry
☒	☐	MRI-based neuroimaging

Human research participants

Policy information about studies involving human research participants

Population characteristics	The characteristics of the cohort is described in table 1. The cohort contained 2315 CCS out of which 2145 are currently alive. The gender distribution was 49,7% males and 50,3% females. We had information of the primary childhood cancer diagnosis as sorted into 12 established diagnoses groups. As controls, 5 persons per childhood cancer survivor were drawn from the general population based on sex, year of birth and place of residency. A control occurs only once in the cohort and can only be used to match one single CCS.
Recruitment	Treatment data and information on primary childhood cancer diagnosis was extracted from a quality registry for all persons who had survived passed 5 years from childhood cancer diagnosis. This is described in figure 1. The law governing quality registries does not impose a requirement of consent from the individual. At the time of diagnosis, the parents of the patients were informed and presented with the possibility of opting out from several quality registries. All data in the project was pseudonymized to allow identification by health care services in case novel life-threatening late effects were identified.
Ethics oversight	The ethical organ KVB at the Council of Skåne approved the extraction and pairing to outcome data from the National Health and Welfare Board. The Ethical Review Board approved the study as a whole (permit number 2018-022).

Note that full information on the approval of the study protocol must also be provided in the manuscript.

Clinical data

Policy information about clinical studies

All manuscripts should comply with the ICMJE guidelines for publication of clinical research and a completed CONSORT checklist must be included with all submissions.

Clinical trial registration	This was not a clinical trial.
Study protocol	This was not a clinical trial.
Data collection	Data was collected into the quality registry from 2005-2017 and the details are described in Wiebe et al Eur J Epidemiol. 2018 Nov;33(11):1125-1129. doi: 10.1007/s10654-018-0437-1. Epub 2018 Sep 6. PMID: 30191362. The data collection for the current study is outlined in Figure 1 of the manuscript.
Outcomes	The outcome data came from the national death registry, the outpatient setting and in-patient setting. We included both the outpatient setting and in-patient setting as sources of outcomes, as the aim of the study was to identify novel associations between childhood cancer, its treatment and any reported outcomes.
